# Serial T2-Weighted Magnetic Resonance Images Acquired on a 1.5 Tesla Magnetic Resonance Linear Accelerator Reveal Radiomic Feature Variation in Organs at Risk: An Exploratory Analysis of Novel Metrics of Tissue Response in Prostate Cancer

**DOI:** 10.7759/cureus.4510

**Published:** 2019-04-20

**Authors:** Joshua W Lorenz, Diane Schott, Lisa Rein, Farshad Mostafaei, George Noid, Colleen Lawton, Meena Bedi, X. A Li, Christopher J Schultz, Eric Paulson, William A Hall

**Affiliations:** 1 Radiation Oncology, Medical College of Wisconsin, Milwaukee, USA; 2 Biostatistics, Medical College of Wisconsin, Milwaukee, USA

**Keywords:** prostate cancer, toxicity, magnetic resonance linear accelerator, radiomics, personalized medicine, image-guided radiation therapy, tumor targeting, organs at risk

## Abstract

"Delta-radiomics" investigates variations in quantitative image metrics over time and can yield important clinical information. We hypothesized that in patients undergoing active radiation therapy (RT) for prostate cancer (PCa), there would exist observable variation in the quantitative metrics that describe the T_2_-weighted (T_2W_) intensity histogram in the prostate and surrounding organs at risk (OAR) over time. We investigated the feasibility of acquisition and subsequent analysis of the delta-radiomic profiles of these regions of interest (ROI) in serial T_2W _magnetic resonance (MR) images obtained on a 1.5 Tesla (T) Magnetic Resonance Linear Accelerator (MRL). Principally, we sought to illustrate the significance of longitudinal radiomic data acquisition for tissue response monitoring and provide a framework for future hypothesis driven research.

Patients with PCa undergoing treatment with RT were compiled from an ongoing prospective observational imaging trial using a 1.5 T MRL (NCT30500081). Contiguous axial slices of prostate parenchyma were contoured and temporally normalized to sections of Sartorius muscle which served as a control. Similarly, contiguous sections of rectal and bladder wall adjacent to the prostate were contoured and temporally normalized to regions of these organs further removed from the planning target volume (PTV). First order statistical descriptors of the T_2W _intensity histogram were extracted and evaluated for changes over time using linear mixed effects regression modeling and post-hoc contrasts. Benjamini-Hochberg corrections were employed to reduce the effects of multiple testing and control for the false discovery rate (FDR).

Four patients with a median age of 69 comprised this exploratory cohort. One patient had low-risk disease, two had intermediate (one favorable, one unfavorable), and one had high risk disease. Three out of four patients underwent definitive radiation to 75.6 Gray (Gy) in 42 fractions and one received hypofractionated therapy to a total dose of 70 Gy over 28 fractions, and all received treatment on a conventional linear accelerator. The most significant acute toxicity event was grade 2 GU dysfunction observed in two patients. Follow up ranged from 1 month to 10 months post treatment, and no long-term complications were reported in patients who completed treatment at least one month prior. Bladder wall adjacent to the prostate demonstrated significant variation in the mean and median metric values after the first week of treatment. In addition, rectal wall adjacent to the prostate exhibited significant variation in the mean, median, and standard deviation metric values by the second week of treatment. No significant variation in any radiomic feature was observed in the Sartorius control.

This exploratory study is one of the earliest examining the delta-radiomic characteristics of the T_2W_ intensity histogram in OAR extracted from images acquired on a 1.5 T MRL in patients actively being treated with RT for PCa. We demonstrated a feasible approach to longitudinal radiomic data acquisition providing limitless opportunity for future research. Analysis of the delta-radiomic profiles in OAR revealed significant variation in metrics after only one week of RT in bladder and rectal wall adjacent to the prostate. These findings must be further investigated and validated with expanded data sets with long-term follow up and correlation to clinical outcomes including toxicity and tumor control.

## Introduction

Prostate cancer (PCa) is the most common malignancy and the second leading cause of cancer-related death in men with 118.2 incident cases and 19.5 attributable deaths per 100,000 persons per year [1]. This disease presents unique oncological challenges in the initial assessment, risk stratification, as well as personalization of treatment. Radiation therapy (RT) offers a minimally invasive, and curative treatment modality. Attempts to improve disease control efficacy of RT in PCa have focused on dose escalation, increased target conformality, the inclusion of androgen deprivation therapy, and adjuvant chemotherapy [2-4]. These approaches have led to significant improvements in disease control. However, despite improved conformality and application of rigid dose constraints, some patients continue to experience significant toxicity events secondary to radiation-induced damage to the bladder or rectum. There is a dire need for objective and accessible metrics physicians can utilize to assess patients for the risk of dose-limiting toxicity before or during RT. Such metrics would facilitate more effective patient counselling and enable early treatment adaption or therapeutic intervention. Despite the conceivable benefits this data would provide, there exists a paucity of research into such measures.

Radiomics, via high-throughput software assisted mining of quantifiable image data, provides the infrastructure for the identification of imaging biomarkers through quantification of both macroscopic and microscopic tumor or normal tissue characteristics [5]. Macroscopic imaging biomarkers include the size, shape, and volumetric measurements of the tumor whereas microscopic imaging biomarkers describe features including textural patterns (signal intensity, heterogeneity) or dynamic perfusion characteristics obtained from diffusion weighted magnetic resonance (MR) sequences [6]. The advantage RT and radiomics together provides is the ability to repeatedly and noninvasively capture qualitative and quantitative data in a longitudinal manner [7]. MR imaging presents a particularly attractive option for longitudinal radiomic assessment due to its enhanced spatial resolution and soft tissue delineation capabilities. A variety of clinical applications utilizing MR-based radiomic analysis have been developed. One tool differentiates glioblastoma pseudoprogression from true progression while another predicts the probability of pathologic complete response (pCR) after neoadjuvant chemoradiotherapy in patients with locally advanced rectal cancer [8-9].

Much of the current literature involving MR-based radiomic analysis focuses on feature analysis before and after treatment. The understanding and utilization of inter-treatment metric variations, or “delta-radiomics”, remains poor in patients undergoing RT for PCa, yet presents a novel area for research. The advent of linear accelerators equipped with on onboard MR imagers will both improve targeting capabilities and allow for adaptive, real-time treatment planning [10-11]. Magnetic Resonance Linear Accelerators (MRLs) may also enable feasible and convenient radiomic feature acquisition from organs at risk (OAR) while patients are undergoing RT. Acquisition of such metrics may improve treatment response monitoring including assessment of radiation induced changes in both malignant tissue and OAR.

We present a series demonstrating the feasibility of acquisition and analysis of the delta-radiomic profiles of OAR for patients undergoing RT for PCa using images acquired on a 1.5 Tesla (T) MRL. Moreover, we propose potential future directions for similar research that may further personalize and improve clinical outcomes for patients.

## Materials and methods

Patient selection, imaging acquisition, and contouring

A total of four patients undergoing intensity-modulated radiotherapy (IMRT) for PCa were compiled from an ongoing prospective observational imaging clinical trial using an Elekta 1.5 T MRL (NCT30500081). All patients provided informed consent for weekly imaging using the MRL while they were undergoing a course of RT on a conventional linear accelerator. Images were obtained between November 2017 and March 2018. Per protocol, subjects were immobilized with pillows, cushions and/or other restraining devices while on the MRL, in the same way, they were in the treatment room.

Using the software MIM, (MIM version 6, MIM Software Inc.; Beachwood, OH), serial 3-dimensional (3D) T2-weighted (T2W) MR scans were collected from one week prior to the initiation of treatment and weekly thereafter. T2W sequences were used for their ability to clearly distinguish rectal and bladder walls from surrounding tissue, which can be difficult on other commonly used MR sequences. Contiguous 3 millimeter (mm) axial slices of the prostate as well as rectal and bladder wall in immediate proximity to the prostate were contoured. The region of interest (ROI) labelled “prostate” was defined as and contoured beginning in the inferior plane of the pubic symphysis and extending 2.4 centimeters (cm) superiorly to encompass the prostatic parenchyma and any lesion contained in that volume. This border captured nearly the entire prostatic parenchyma and standardized one dimension of the ROI volume.

The ROI labelled “prostatic rectum” was defined as and contoured beginning in the inferior plane of the pubic symphysis and extending 2.4 cm superiorly following the contour of the anterior rectal wall. This border value consistently encompassed the highest dose region posterior to the prostate. Subsequently, 2.4 cm of the anterior rectal wall superior to “prostatic rectum” were contoured and defined as the ROI labelled “rectum.” The lateral extent of both of these ROI mirrored the lateral extent of the “prostate.”

The ROI labelled “prostatic bladder” was defined as and contoured beginning in the plane of the base of the prostate and extending 1.2 cm superiorly. This distance consistently encompassed the highest dose region superior to the prostate and was also reproducible (4 slices above and 4 slices below the center of the prostate gland). The anterior border of this volume mirrored the anterior border of the “prostate” and encompassed all visible inferoposterior portions of the bladder wall. Anterior to the anterior extent of the “prostatic bladder,” an additional 1.2 cm slices of bladder wall were contoured and labelled “bladder,” encompassing all visible anterosuperior portions of the bladder wall. The superior borders of both “prostatic bladder” and “bladder” mirrored the superior border of “rectum.”

**Figure 1:**
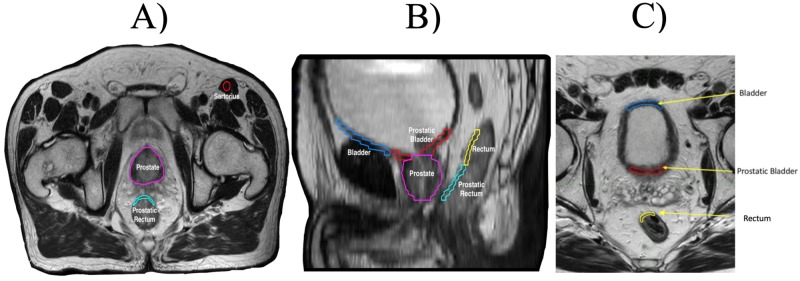
Examples of contours of the ROI in the A) axial and B) sagittal planes. Panel C) represents an example of the equivalency of the superior borders of the bladder, prostatic bladder, and rectum. Regions of the bladder and the rectal wall adjacent to the prostate were labelled "prostatic bladder" or "prostatic rectum" while regions of these OAR further removed from the PTV were labelled "bladder" and "rectum" ROI: Region(s) of Interest; OAR: Organ(s) at Risk; PTV: Planning Target Volume

Axial slices of sartorius muscle were contoured to serve as a control and to conduct “test re-test” evaluation of calculated radiomic features. This ROI was labelled “muscle.” All contours were visually inspected by an attending board-certified radiation oncologist, in conjunction with the patient’s treatment plan, to ensure consistency and modified if necessary. Examples of these contours and labels can be seen in Figure 1.

Quantitative feature extraction

MIM software was used to extract quantitative imaging features. The quantitative features extracted included: max-to-mean ratio, kurtosis, mean, median, skewness, and standard deviation. These are first-order statistical descriptors based on the average voxel values and calculated from the MIM statistical libraries. We hypothesized that as the tissues received increasing RT dose, there would exist changes in the proportion of tissue distributions (functioning vs. nonfunctioning, undamaged vs. damaged) within that tissue, leading to identifiable variations in the quantitative features contained within the OAR volumes. These changes will result in changes in the average, asymmetry, and degree of outliers (or pointedness) which are quantified by mean, skewness, and kurtosis respectively.

Statistical analysis 

For each tissue, MR features were summarized using the mean, standard deviation, median, and range at each timepoint. The frequency of missing data was reported, and missing observations were excluded from the analysis. Linear mixed effects regression modeling was performed to compare MR features over time. Each continuous MR feature (mean, median, standard deviation, skewness, kurtosis, and max-to-mean ratio) was modeled separately for each tissue (muscle, normal bladder, prostatic bladder, normal rectum, prostatic rectum, and prostate). Each model included a random patient-specific intercept and time (discrete) as a predictor. Post-hoc contrasts were used to compare each time-point to baseline (week 2 vs. 1, week 3 vs. 1, and week 4 vs.1).

As a secondary analysis, the linear mixed effects modeling was repeated for each ROI after dividing by the contemporaneous measurements for muscle tissue; the rationale for this was to normalize the experimental data to a reference tissue in each individual patient that was receiving a (relatively) minimal amount of RT dose, and thus should not be demonstrating radiomic changes over time. Similarly, the regression analysis was repeated for “prostatic bladder” after dividing by the “bladder” values, and “prostatic rectum” after dividing by the “rectum” values. All statistical analyses were performed using R version 3.3.4 (R Foundation for Statistical Computing, http://www.R-project.org). All p-values were 2-sided and p less than 0.05 was considered statistically significant. Corrections for multiple testing were performed using the Benjamini-Hochberg procedure which controls the false discovery rate (FDR) [12]. The Benjamini-Hochberg was selected as this correction is less conservative than Bonferroni for the purposes of this exploratory analysis. In addition, the interdependence of these variables made Bonferroni corrections perhaps overly conservative.

## Results

A total of four patients were accrued to this prospective observational imaging trial and underwent between two to four MR scans during treatment. Each scan acquired on the MRL was well tolerated. The median age was 69 years. One patient had low-risk disease, two had intermediate (one favorable, one unfavorable), and one had high-risk disease. Three out of four patients underwent definitive radiation to 75.6 Gy in 42 fractions. One received hypofractionated therapy to a total dose of 70 Gy over 28 fractions. Patient demographics can be seen in Table 1. 

**Table 1:** Patient demographics and clinical characteristics iPSA: Initial Prostate Specific Antigen level

Patient	Age	Gleason Score	iPSA (ng/mL)	Clinical Stage	Dose (cGy)	Dose per Fraction (cGy)	Number of Fractions	Weeks of Scan
1	73	3+4=7	7.2	T2aN0M0	7,000	250	28	1, 2, 3
2	63	3+3=6	4.7	T2aN0M0	7,560	180	42	1, 4
3	66	4+5=9	7.3	T3aN0M0	7,560	180	42	1, 2, 3, 4
4	72	3+4=7	6.1	T2cN0M0	7,560	180	42	1, 2, 4

Quantitative features extracted from the control ROI “Sartorius” can be seen in Table 2. Based on the evaluation of IMRT plans, the total dose to the Sartorius muscle was less than 5 Gy. There were no significant week-to-week changes identified in the radiomic features analyzed in this ROI. As such, there can be a greater degree of confidence that the observed metric variations in the other ROI volumes reflect tissue changes secondary to a higher degree of dose spill from the PTV rather than daily fluctuations resulting from the variety of factors that can affect T2W MR intensity.

**Table 2:** Delta-radiomic profiles of extracted T2W intensity histogram metrics from the control ROI, "Sartorius" ROI: Region(s) of Interest; T2W: T2-weighted

T_2W _Intensity Histogram Metric	Calculated Metric Specific Statistical Descriptors	Week 1	Week 2	Week 3	Week 4
Mean	Mean	25.55	25.54	25.02	28.1
	SD	2.63	4.74	8.34	10.37
	Median	25.43	25.81	25.02	26.17
	Min & Max	22.48, 28.88	20.67, 30.14	19.12, 30.92	18.82, 30.92
Median	Mean	24.17	24.96	23.28	26.92
	SD	2.4	5.06	6.61	9.66
	Median	24.26	25.5	23.28	26.25
	Min & Max	21.16, 27.01	19.65, 29.72	18.60, 27.95	17.61, 36.90
Standard Deviation	Mean	8.02	6.7	8.94	9.54
	SD	1.3	0.37	5.1	6.18
	Median	7.68	6.67	8.94	6.02
	Min & Max	6.89, 9.83	6.35, 7.09	5.33, 12.55	5.92, 16.67
Skewness	Mean	1.06	0.93	1.11	0.84
	SD	0.51	0.41	0.62	0.28
	Median	0.89	0.95	1.11	0.94
	Min & Max	0.69, 1.79	0.50, 1.32	0.67, 1.55	0.52, 1.06
Kurtosis	Mean	2.18	1.91	1.69	1.64
	SD	1.94	0.75	1.23	0.55
	Median	1.61	1.84	1.69	1.44
	Min & Max	0.59, 4.91	1.19, 2.69	0.82, 2.56	1.22, 2.26
Max-to-Mean Ratio	Mean	2.5	2.23	2.6	2.67
	SD	0.32	0.44	76	0.37
	Median	2.53	2.12	2.6	2.88
	Min & Max	2.01, 2.86	1.86, 2.72	2.07, 3.14	2.24, 2.88
N		4	3	2	3_2_

The results of the mixed-effects linear regression modeling for “prostatic bladder” and “prostatic rectum” temporally normalized to regions of these OAR further removed from the PTV can be seen in Table 3. Analysis of these regions’ delta-radiomic profiles revealed several statistically significant findings. In “prostatic bladder”, the mean and median both exhibited statistically significant changes, both before and after the Benjamini-Hochberg corrections were performed. In addition, “prostatic rectum" exhibited statistically significant changes in the mean, median, and standard deviation. These remained significant following the Benjamini-Hochberg corrections. Patient follow up post RT ranged from 1 month to 10 months. The highest grade of acute toxicity was grade 2 GU toxicity. Patient toxicity and follow-up data can be seen in Table 4.

**Table 3:** Mixed effects linear regression modeling of intra-patient week-to-week variation in T-2 weighted intensity histogram metrics of the bladder and rectal wall adjacent to the prostate normalized to regions of these organ(s) at risk further removed from the planning target volume

ROI	T_2W_ Intensity Histogram Metric	Estimated Intra-Patient Variation [95% Confidence Interval]					
		Week 2 vs. Week 1	p-value	Week 3 vs. Week 1	p-value	Week 4 vs. Week 1	p-value
Prostatic Bladder	Mean	0.15 [0.00, 0.30]	0.047	0.07 [-0.11, 0.24]	0.381	0.33 [0.19, 0.48]	0.002
	Median	0.19 [0.06, 0.32]	0.015	0.10 [-0.06, 0.26]	0.159	0.41 [0.28, 0.54]	<0.001
	Standard Deviation	0.18 [-.018, 0.53]	0.261	0.27 [-0.15, 0.68]	0.157	0.05 [-0.30, 0.40]	0.734
	Skewness	3.93 [-2.59, 10.44]	0.182	3.21 [-4.28, 10.71]	0.320	2.54 [-3.97, 9.05]	0.362
	Kurtosis	-0.99 [-4.92, 2.94]	0.545	3.12 [-1.41, 7.64]	0.137	2.14 [-1.78, 6.06]	0.220
	Max-to-Mean Ratio	0.13 [-0.11, 0.38]	0.220	0.03 [-0.26, 0.32]	0.793	0.18 [-0.06, 0.43]	0.116
Prostatic Rectum	Mean	0.18 [0.06, 0.30]	0.012	0.18 [0.04, 0.32]	0.022	0.10 [-0.02, 0.22]	0.079
	Median	0.23 [0.10, 0.35]	0.005	0.19 [0.04, 0.34]	0.020	0.10 [-0.03, 0.22]	0.101
	Standard Deviation	0.11 [-0.25, 0.46]	0.468	0.50 [0.10, 0.90]	0.024	0.10 [-0.25, 0.46]	0.491
	Skewness	0.47 [-2.34, 3.29]	0.684	0.37 [-2.83, 3.56]	0.780	2.59 [-0.22, 5.40]	0.064
	Kurtosis	-0.88 [-3.24, 1.48]	0.380	-0.55 [-3.27, 2.17]	0.624	-1.62 [-3.98, 0.73]	0.137
	Max-to-Mean Ratio	0.14 [-0.08, 0.37]	0.159	0.15 [-0.11, 0.41]	0.191	0.14 [-0.08, 0.37]	0.166

**Figure 2:**
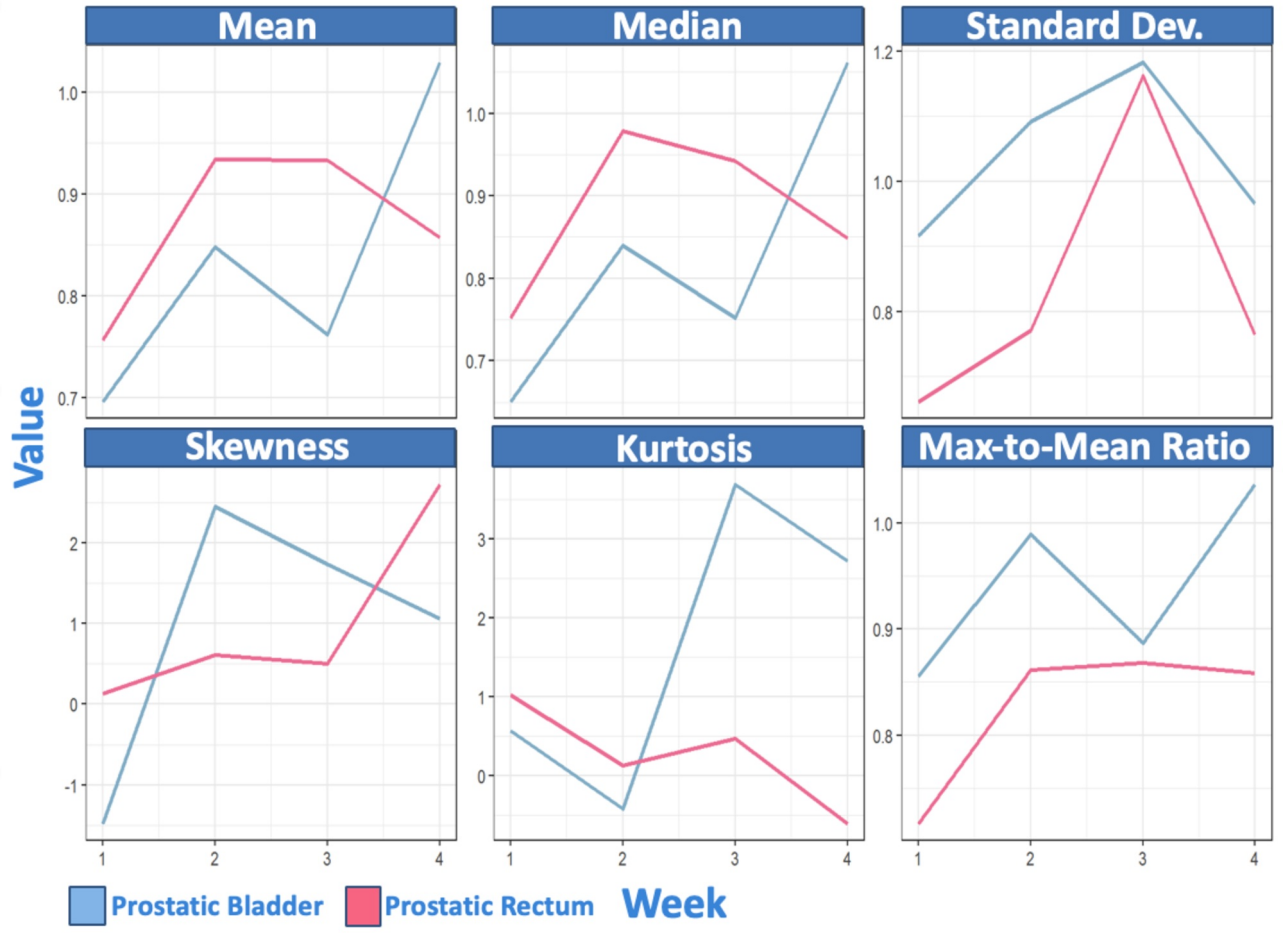
Linear mixed model estimate plots of the changes in T-2 weighted intensity histogram metric values in "prostatic bladder" and "prostatic rectum" temporally normalized to regions of these organ(s) at risk further removed from the planning target volume

Linear mixed model estimate plots of the variation in T_2W_ metric values over time in "prostatic bladder" and "prostatic rectum" can be seen in Figure 2. 

Patient follow up post RT ranged from 1 month to 10 months. The highest grade of acute toxicity was grade 2 GU toxicity. Patient toxicity and follow-up data can be seen in Table 4.

**Table 4:** Acute and long-term toxicity events of patients at last follow-up GU: Genitourinary; GI: Gastrointestinal

Patient	Acute GU Toxicity	Acute GI Toxicity	Long Term GU Toxicity	Long Term GI Toxicity	Last Follow Up
1	Grade 1	Grade 1	None	None	10 months
2	Grade 2	Grade 1	None	None	9 months post
3	Grade 1	Grade 1	None	None	8 months post
4	Grade 2	Grade 1	--	--	1 month post

## Discussion

The introduction of the MRLs and the advancing field of radiomics offers extraordinary opportunity for basic, translational, and clinical research. Together, these tools will help solve questions that will revolutionize RT, including the best methods to monitor normal and malignant tissues’ response to RT. The superior soft tissue delineation afforded by MRLs paired with longitudinal radiomic feature acquisition offers an innovative method of tissue response assessment during RT for PCa. That is to say, tissues in OAR may exhibit changes in radiomic features early on during a course of treatment that correlates with the development of clinically meaningful events. If the significance of these changes can be elucidated, action to modify plans to mitigate toxicity in these tissues could be taken preemptively. The collection of such data in a longitudinal manner is ideally suited for the MRL which captures real-time, intra-treatment MR images. This capability is unprecedented in the field of radiation oncology and warrants additional hypothesis-driven research and long-term validation. 

Research consortiums have already been established to expand the understanding and use of radiomics to improve patient outcomes [13]. One of the earliest publications in this realm was authored by Aerts et al. who identified the prognostic power of pre-treatment computed tomography (CT) based tumor imaging biomarkers (TIBs) in both non-small cell lung cancer (NSCLC) and head and neck tumors [7]. This seminal work in 2014 led to several publications examining similar pre-treatment assessments of radiomic features associated with potential tumor response [13-15]. Important to note is that these early radiomic analysis publications did not capture longitudinal data to describe delta-radiomic profiles. In fact, the majority of current research combining radiomics and oncologic therapy thus far has focused solely on the correlation of pre-treatment radiomic metric values of the target lesion to oncologic outcomes rather than longitudinal monitoring of normal tissue response to RT. The latter’s importance cannot be understated and demands further exploration as delays or early cessation from RT secondary to normal tissue toxicity can sacrifice tumor control and other oncologic outcomes [16]. The lack of clinical investigation in this arena may be secondary to the incredibly complex and tedious nature of collecting many of the available radiomic metrics using published methods that rely on advanced computing power.

A recent publication that did employ intra-treatment image acquisition and radiomic feature analysis was authored by Paul et al [17]. In this series daily CT scans acquired during RT for lung cancer were used to measure changes in Hounsfield units with the intention of correlating these changes with patient survival. In addition, CT-based radiomic features have also been used to measure tumor response during a course of treatment with chemoradiation therapy in pancreatic cancer [18]. The ability to objectively measure patient-specific tissue responses during a course of RT represents a powerful opportunity to personalize and adapt treatment based on those measured responses as well as identify patients with tumors responding well, or poorly, to RT. To take full advantage of the insights longitudinal radiomic analysis could provide, greater emphasis should be placed on identifying and implementing practical methods for radiomic feature collection during RT that physicians can utilize to aid in real-time treatment decisions. 

The series presented offers one of the first, to our knowledge, to demonstrate the feasibility of delta-radiomic profile acquisition and analysis of OAR surrounding the prostate obtained from MR images acquired on a 1.5 T MRL in patients undergoing active RT for PCa. This exploratory analysis serves purely to illuminate methodology and advocate for the potential benefits that longitudinal radiomic data acquisition could offer, specifically in regard to normal tissue response monitoring. Furthermore, this manuscript functions as a conceivable framework for future hypothesis-driven clinical research by demonstrating a practical method for radiation oncologists to acquire a few common radiomic metrics using widely available commercial tools. It is important to emphasize such metrics could easily be collected during weekly reviews as a method of objectively assessing normal organ response. We demonstrated that such data can be feasibly and quickly extracted using technology and techniques familiar to radiation oncologists. Furthermore, our analysis of the delta-radiomic profiles of the OAR surrounding the prostate demonstrated statistically significant changes in several metrics occur early on during a treatment. There was no significant variability in radiomic features measured in a control structure (Sartorius muscle). This provided valuable test re-test data demonstrating the reproducibility of these quantitative metrics. These findings support the hypothesis that the radiomic changes in the high dose regions of the rectum and bladder may indeed be secondary to RT given the absence of changes in a muscular control that received much lower doses of radiation. At this stage in the analysis, it is unclear why some metrics in the ROI display early vs. delayed or transient vs. preserved significant week-to-week differences. A potential explanation is that the observed radiomic metrics reflect differences in the immunophenotype of the tissues in response to radiation [19]. Definitive conclusions regarding the T2W radiomic metric changes in OAR surrounding the prostate cannot be drawn from this series. However, what should be emphasized is the novelty of concept and feasibility of the methods intended to promote future research efforts in larger cohorts of patients. If these findings could be replicated in a larger cohort of patients and subsequently shown to correlate with clinically significant toxicity endpoints, they could offer an entirely novel objective metric of normal tissue response and toxicity assessment in patients undergoing RT for PCa. 

There are limitations to this series that are important to note. This is a small and exploratory cohort consisting of only four patients. Not all patients in the cohort received homogenous dose prescription and there were missing MR scan data points. Follow-up only ranged to 10 months which is not long enough to evaluate for late clinically relevant events. This series requires expansion into a much larger cohort with more extensive data collection and long-term follow-up for validation and correlation to clinical outcomes including toxicity and tumor control.

Despite these limitations, these prospectively collected metrics do highlight the potential novelty of the soft tissue response assessment capabilities of a 1.5 T MRL. Moreover, this methodology represents a quick and feasible approach for radiation oncologists to collect and study both normal and malignant tissue imaging biomarkers which could, through continued investigation, assist in the prediction of clinically significant endpoints. There are extensive possibilities for future inquiry that could follow similar methods outlined in this manuscript in PCa or many other tumor sites and anatomic regions. Our future efforts will focus on series expansion and correlating quantitative findings with clinical endpoints and histopathological outcomes. 

## Conclusions

This exploratory study is one of the earliest examining the delta-radiomic characteristics of the T_2W_ intensity histogram in OAR extracted from images acquired on a 1.5 T MRL in patients actively being treated with RT for PCa. Our analysis of the delta-radiomic profiles in OAR revealed significant variation in metrics after only one week of RT in the bladder and the rectal wall adjacent to the prostate. These preliminary and nonconclusive results are intended to present a methodology that must be further investigated and validated with expanded data sets with long-term follow up and correlation to clinical outcomes including toxicity and tumor control. Furthermore, we demonstrated a feasible approach to longitudinal radiomic data acquisition providing limitless opportunity for future research while simultaneously reporting existing radiomic data and emphasizing potential future benefits of continued investigation.
